# Supplementary material for: Risk of New-Onset Diabetes Mellitus Associated with Antirheumatic Drugs in Patients with Rheumatoid Arthritis: A Nationwide Population Study
Source: J Clin Med. 2022 Apr 10;11(8):2109. doi: 10.3390/jcm11082109 (PMC9026381; doi:10.3390/jcm11082109)
Supplement: Supplementary file 1 [file jcm-11-02109-s001.zip › jcm-1652406-supplementary.pdf]

Supplement Table S1. Operational definition of the exclusion criteria, comorbidities, and individual drug of comedications.

Exclusion/comorbidities	ICD-10 codes/National Health Insurance Payment codes
Prior diagnosis of DM	R81, E10-E14, O24 or previous anti-diabetic drug prescription
HIV infection	B20-24, R75, and Z21
Dialysis	National Health Insurance Payment codes (O701x, O702x, O703x, O704x, O705x, O706x, O707x, and O708x)
Interstitial lung disease	J84
Cancer diagnosis	C00-97 and cancer registration codes (V027, V193, and V194)
Solid organ transplantation	Z940, Z941, Z942, Z943, Z944, and Z948
Other connective tissue diseases including systemic lupus erythematosus, and mixed connective tissue disease	M06-09, M30-36, M45, M468, M4691, M4699, and M6099
Ischemic heart diseases	I20–25
Congestive heart failure	I099, I110, I130, I132, I255, I42, I43, and I50
Peripheral vascular disease	I70, I71, I731, I738, I739, I771, I790, I792, K551, K558, K559, Z958, and Z959
Cerebrovascular disease	I60, I61, I62, I63, I64, I65, I66, I67, I68‡, and I69
Dementia	F00, F01, F02, F03, F051, G30, and G311
Carotid stenosis	I65 and I74
Ischemic stroke	I63, I64, I693, I694, and G459
Chronic pulmonary disease	I278, I279, J40, J41, J42, J43, J44, J45, J46, J47, J60, J61, J62, J63, J64, J65, J66, J67, J684, J701, and J703
Peptic ulcer disease	K25, K26, K27, and K28
Mild liver disease	B18, K73, K74, K700, K701, K702, K703, K709, K713, K714, K715, K717, K760, K762, K763, K764, K768, K769, and Z944
Moderate to severe liver disease	I850, I859, I864, I982, K704, K711, K721, K729, K765, K766, and K767
Diabetes without complications†	E100, E101, E106, E108, E109, E110, E111, E116, E118, E119, E120, E121, E126, E128, E129, E130, E131, E136, E138, E139, E140, E141, E146, E148, and E149
Diabetes with complications	E102, E103, E104, E105, E107, E112, E113, E114, E115, E117, E122, E123, E124, E125, E127, E132, E133, E134, E135, E137, E142, E143, E144, E145, and E147
Hemiplegia	G81, G82, G041, G114, G801, G802, G830, G831, G832, G833, G834, and G839
Any tumor including leukemia and lymphoma	C00–C26, C30–C34, C37–C41, C43, C45–C58, C60–C76, C81–C85, C88, and C90–C97,
Metastatic solid tumor	C77, C78, C79, and C80
Hypertensive diseases	I10–I15
Dyslipidemia	E78
Asthma	J45 and J46
COPD	J44
Moderate to severe renal failure	I120, I131, N032–N037, N052–N057, N18, N19, N250, Z490–Z492, Z940, and Z992

Comedications	Individual drugs
DM medications	Human insulin
	Insulin glulisine
	Insulin lispro
	Insulin aspart
	Insulin glargine
	Insulin determir
	Exenatide
	Liraglutide
	Lixisenatide
	Albiglutide
	Dulaglutide
	Metformin
	Glibenclamide
	Glipizide
	Gliquidone
	Gliclazide
	Glimepiride
	Acarbose
	Miglitol
	Voglibose
	Rosiglitazone
	Pioglitazone
	Lobeglitazone
	Sitagliptin
	Vildagliptin
	Saxagliptin
	Alogliptin
	Linagliptin
	Gemigliptin
	Anagliptin
	Evogliptin
	Teneligliptin
	Repaglinide
	Nateglinide
	Mitiglinide
	Dapagliflozin
	Canagliflozin
	Empagliflozin
	Ipragliflozin
	Ertugliflozin
Statin	Simvastatin
	Lovastatin
	Pravastatin
	Fluvastatin
	Atorvastatin
	Rosuvastatin
	Pitavastatin

Glucocorticosteroids	Prednisolone<br>Methylprednisolone<br>Triamcinolone<br>Deflazacort
Conventional DMARDs	Methotrexate<br>Hydroxychloroquine<br>Sulfasalazine<br>Leflunomide<br>Tacrolimus
Biologic DMARDs	Etanercept<br>Infliximab<br>Adalimumab<br>Certolizumab<br>Golimumab<br>Abatacept<br>Rituximab<br>Tocilizumab
Targeted synthetic DMARDs	Tofacitinib<br>Baricitinib

DM diabetes mellitus, *HIV* human immunodeficiency virus, *COPD* chronic obstructive pulmonary disease, *DMARDs* disease-modifying antirheumatic drugs

Supplement Table S2. Association between cumulative use of DMARDs and incident diabetes in patients with RA

	**Case[a] (*n* = 3,772)**	**Control[a] (*n* = 14,830)**	**Unadjusted OR (95% CI)**	***p* value**	**Adjusted OR[b] (95% CI)**	***p* value**
			Hydroxychloroquine			
Nonuser	2,143 (56.8)	8,151 (55.0)	1.00	<0.001	1.00	<0.001
1–90 days	338 (9.0)	769 (5.2)	1.78 (1.54–2.05)	<0.001	1.45 (1.23, 1.70)	<0.001
91–180 days	267 (7.1)	793 (5.3)	1.33 (1.15–1.55)	<0.001	1.15 (0.97, 1.36)	0.116
181–270 days	194 (5.1)	889 (6.0)	0.84 (0.71–0.99)	0.034	0.81 (0.68, 0.97)	0.024
271–365 days	830 (22.0)	4,228 (28.5)	0.73 (0.67–0.80)	<0.001	0.76 (0.69, 0.84)	<0.001
			Methotrexate			
Nonuser	1,013 (26.9)	4,199 (28.3)	1.00	<0.001	1.00	<0.001
1–90 days	330 (8.7)	686 (4.6)	2.18 (1.87–2.55)	<0.001	1.59 (1.34, 1.89)	<0.001
91–180 days	230 (6.1)	773 (5.2)	1.29 (1.09–1.52)	0.003	0.96 (0.80, 1.15)	0.630
181–270 days	294 (7.8)	1,109 (7.5)	1.12 (0.96–1.30)	0.144	0.92 (0.78–1.08)	0.296
271–365 days	1,905 (50.5)	8,063 (54.4)	0.96 (0.88–1.04)	0.313	0.81 (0.74–0.89)	<0.001
			Leflunomide			
Nonuser	2,448 (64.9)	10,732 (72.4)	1.00	<0.001	1.00	<0.001
1–90 days	196 (5.2)	387 (2.6)	2.28 (1.90–2.73)	<0.001	1.56 (1.29–1.90)	<0.001
91–180 days	160 (4.2)	399 (2.7)	1.77 (1.47–2.14)	<0.001	1.36 (1.10–1.66)	0.004
181–270 days	182 (4.8)	502 (3.4)	1.61 (1.35–1.92)	<0.001	1.09 (0.90–1.32)	0.402
271–365 days	786 (20.8)	2,810 (18.9)	1.24 (1.13–1.36)	<0.001	0.94 (0.85–1.05)	0.250
			Sulfasalazine			
Nonuser	2,968 (78.7)	11,992 (80.9)	1.00	<0.001	1.00	<0.001
1–90 days	244 (6.5)	460 (3.1)	2.26 (1.92–2.67)	<0.001	1.56 (1.30–1.87)	<0.001
91–180 days	127 (3.4)	358 (2.4)	1.47 (1.19–1.81)	<0.001	1.24 (0.98–1.56)	0.069
181–270 days	80 (2.1)	388 (2.6)	0.85 (0.66–1.09)	0.191	0.85 (0.65–1.11)	0.219
271–365 days	353 (9.4)	1,632 (11.0)	0.87 (0.77–0.98)	0.021	0.94 (0.82–1.07)	0.329
			Tacrolimus			

Nonuser	3,314 (87.9)	13,743 (92.7)	1.00	<0.001	1.00	<0.001
1–90 days	88 (2.3)	174 (1.2)	2.11 (1.63–2.74)	<0.001	1.48 (1.12–1.96)	0.005
91–180 days	88 (2.3)	148 (1.0)	2.49 (1.91–3.26)	<0.001	1.81 (1.36–2.41)	<0.001
181–270 days	63 (1.7)	153 (1.0)	1.74 (1.29–2.35)	<0.001	1.27 (0.92–1.76)	0.139
271–365 days	219 (5.8)	612 (4.1)	1.50 (1.28–1.76)	<0.001	1.27 (1.07–1.51)	0.006

DMARDs disease-modifying antirheumatic drugs, *RA* rheumatoid arthritis, *OR* odds ratio, *CI* confidence interval, *CCI* Charlson Comorbidity Index.

[a]Data are given as number (percentage).

[b]Adjusted for CCI (ordinal), use of steroid (cumulative prescribed dose quintiles), use of statin (yes/no), and use of other DMARDs.
